# Supplementary material for: The causal relationship between the human gut microbiota and pyogenic arthritis: a Mendelian randomization study
Source: Front Cell Infect Microbiol. 2024 Nov 26;14:1452480. doi: 10.3389/fcimb.2024.1452480 (PMC11629706; doi:10.3389/fcimb.2024.1452480)
Supplement: Supplementary file 1 [file DataSheet1.zip › mendelian test/ebi-a-GCST90027820.csv_leave_one_out.pdf]

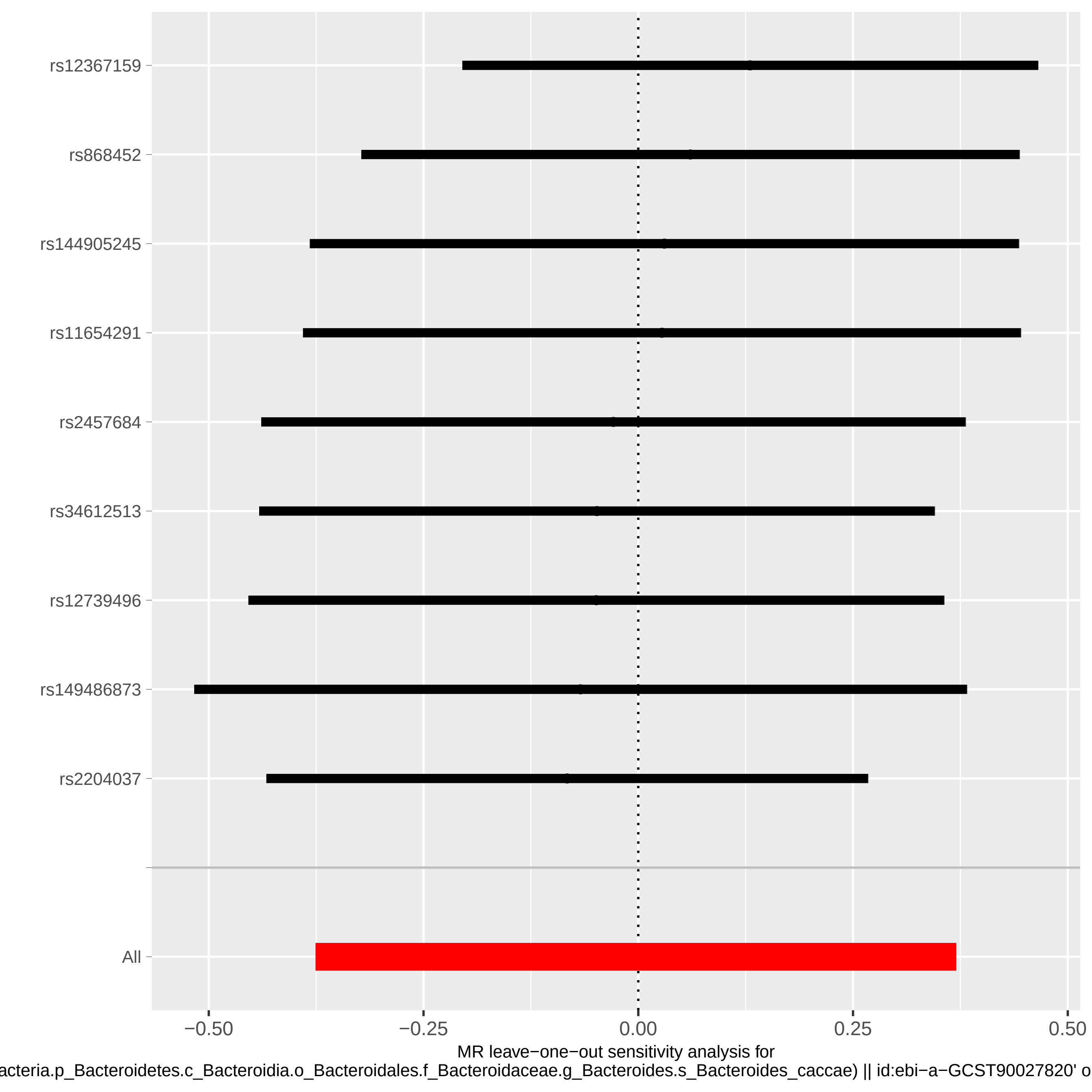

rs12367159
rs868452
rs144905245
rs11654291
rs2457684
rs34612513
rs12739496
rs149486873
rs2204037
All
−0.50
−0.25
0.00
0.25
0.50
MR leave−one−out sensitivity analysis for
acteria.p_Bacteroidetes.c_Bacteroidia.o_Bacteroidales.f_Bacteroidaceae.g_Bacteroides.s_Bacteroides_caccae) || id:ebi−a−GCST90027820' o
